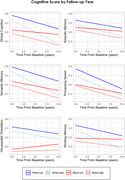# Exploring the Influence of Loneliness on Cognitive Decline: Differential Effects in White and Black Older Adults

**DOI:** 10.1002/alz.088027

**Published:** 2025-01-09

**Authors:** Quinn Lin, Mahsa Dadar, Michael Oliver, Cassandra Morrison

**Affiliations:** ^1^ Carleton University, Ottawa, ON Canada; ^2^ Douglas Mental Health University Institute, Montreal, QC Canada; ^3^ McGill University, Montreal, QC Canada; ^4^ Belmont University, Nashville, TN USA

## Abstract

**Background:**

Approximately 25% of adults 65+ are socially isolated. Social isolation (i.e., loneliness), or the perceived absence of an available and acceptable social network, may be associated with increased cognitive decline. However, there is a limited understanding of how social isolation influences cognitive decline and if this relationship differs as a function of race.

**Methods:**

A total of 3,082 participants (n = 1943 Whites, n = 1139 Blacks) with 20,882 follow‐ups over 10 years were included from the RUSH database. Participants completed the De Jong Gierveld Loneliness Scale and were identified as having either high or low loneliness using a median split of perceived social isolation. Participants were then divided into one of four groups based on race (White vs Black) and loneliness (High vs Low): 1) White‐High (n = 1030), 2) White‐Low (n = 913), 3) Black‐High (n = 493), 4) Black‐Low (n = 646). Linear mixed‐effects models examined group differences in cognitive change over time (global cognition, episodic memory, semantic memory, visuospatial ability, and working memory).

**Results:**

White adults with high loneliness exhibited increased rates of cognitive change compared to low and high loneliness Black adults in all domains (*p*<.01). Compared to White adults with low loneliness, Whites with high loneliness exhibited increased decline in all domains except processing speed and visuospatial ability (*p*<.001). Black adults with high loneliness exhibited increased decline compared to Black adults with low loneliness in all domains (*p*<.01).

**Conclusion:**

Our study reveals a robust association between loneliness and cognitive decline in older adults, with White individuals experiencing a more pronounced impact than their Black counterparts. These findings underscore the need for targeted interventions to address loneliness, particularly among White older adults, to mitigate cognitive decline and promote healthy aging.